# Editorial: Research Methods Pedagogy: Engaging Psychology Students in Research Methods and Statistics

**DOI:** 10.3389/fpsyg.2016.01430

**Published:** 2016-09-21

**Authors:** Lynne D. Roberts

**Affiliations:** School of Psychology and Speech Pathology, Curtin UniversityPerth, WA, Australia

**Keywords:** research methods, statistics, pedagogy, teaching, psychology students, learning

Across disciplines, many introductory research methods students present as uninterested and unmotivated to learn a topic they see as lacking in relevance (Earley, 2014); psychology is no exception (Ruggeri et al., 2008). This is problematic as research methods and statistics are central to the development of professional competence and evidence based psychological practice. Reflecting this, the ability to interpret, design, and conduct basic psychological research forms part of the “scientific inquiry and critical thinking” goal of the *APA Guidelines for the Undergraduate Psychology Major: Version 2.0* (American Psychological Association, 2016), with research methods and statistics a requirement of almost all undergraduate psychology programs (Norcross et al., 2016). Furthermore, the ability to interpret and apply research findings contributes to the development of psychological literacy, the primary outcome of an undergraduate education in psychology (Cranney and Dunn, 2011). This Research Topic brings together current research, innovative evidence-based practice and critical discourse related to engaging undergraduate psychology students in learning quantitative and qualitative methods research.

In the first of fourteen articles, Lacot et al. present a perspective on two methods of stimulating first year undergraduate students' interest in research methods within an introductory psychology course, with the aim of promoting critical thinking about research right from the beginning of the undergraduate degree. Teaching students to critically question what they hear and read, to “evidence check” and propose ways of testing are important components underlying the development of research methods competence.

While interest in research methods can be stimulated in introductory psychology course, most teaching of research methods occurs within dedicated research methods courses. Three articles explore the role of active learning within research methods courses in psychology, a strategy with demonstrated effectiveness for increasing student performance across science, technology, engineering and mathematics courses (Freeman et al., 2014). Allen and Baughman report that in comparison to students in didactic workshops, students in activity based workshops demonstrated greater knowledge of, and confidence in using, research methods, but did not differ in their satisfaction with the learning experience. Rock et al. describe how eLearning systems might be used to actively engage psychology students in research methods and statistics through the application of eLearning pedagogical principles, providing examples of teaching advanced research methods within a virtual world. Lim et al. report that retrieval practice produces better long-term retention of statistical knowledge than does repeated studying. These articles share a focus on the benefits of actively engaging psychology undergraduate students in learning research methods and statistics.

Structural knowledge of research methods can be developed through formal methodology training (Balloo et al., 2016). In an original research article Allen et al. identify a key area of difficulty for psychology students is selecting appropriate statistical tests for analysing data. Provided with a range of vignettes depicting common research scenarios, undergraduate psychology students struggled to articulate a systematic decision making process for selecting statistical tests. This finding highlights the need for a statistical decision making aid based on decision-tree logic to support student learning. Building on this, Allen et al.'s technology report introduces a free mobile app, (Allen et al., 2015), designed to do just that. StatHand guides students through a systematic process for identifying an appropriate statistical test for a wide range of research scenarios, scaffolding learning through a focus on the structural features of research scenarios.

Two further articles focus on the use of technology to support student learning of research methods and statistics. Ellis and Merdian highlight the use of dynamic and interactive data visualizations using the open-source statistical software *R* with a range of packages, such as (Chang et al., 2015), designed for this purpose. These packages enable teachers and students to modify visualizations in real time through, for example, selecting subsets of data to examine and compare, or manipulating inputs. Dynamic, interactive data visualizations have the potential to replace the static graphical illustrations commonly used in teaching research methods statistics. Moreau provides a list of recommended online resources that can be accessed by academics wishing to incorporate dynamic integrative data visualizations into their own teaching of research methods to enhance student learning.

A series of three articles by Perezgonzalez focus on the Null Hypothesis Significant Testing (NHST) controversy as it applies to the teaching of statistics in psychology. Once described as “surely the most bone-headedly misguided procedure ever institutionalized in the rote training of science students” (Rozeboom, 1997, p. 335), NHST remains a commonly used analytic approach in psychology, although now with requirements for the addition of effect sizes, confidence intervals and descriptive text (American Psychological Association, 2009). In the first opinion piece, Perezgonzalez addresses theoretical misinterpretations regarding statistical significance. This is followed by a general commentary article on the use of *p*-values, where he illustrates the differences in interpretation depending upon whether a percentile or probability heuristic is used. The third review article presents a tutorial for teaching hypothesis testing theories. Working from a different perspective Aksentijevic presents statistics anxiety in psychology students as a rational response to myths about the nature of probability and statistics. In his perspective piece Aksentijevic suggests the focus needs to shift away from NHST to the larger role of statistics in research.

Quantitative research methods and statistics predominate in the teaching of research methods within psychology. Perhaps reflecting this, only one article in this research topic focused on teaching qualitative methods. Roberts and Castell examine third year undergraduate psychology students' attitudes to qualitative research. Students viewed qualitative research as a paradigmatic shift requiring new ways of thinking about research that was alternatively construed as a threat or an advantage. Roberts and Castell advocate for the integration of teaching of qualitative and quantitative research methods to reduce the perceived dichotomy between the two.

Rounding off this special issue is an opinion piece on scientific integrity. Schoenherr argues for the explicit inclusion of scientific integrity in the undergraduate psychology curriculum. The importance of this is highlighted by research identifying research misconduct and questionable research practices by psychology students (Rajah-Kanagasabai and Roberts, 2015).

The articles in this research topic contribute to what has been referred to as the “under-researched and under-developed” pedagogical culture for teaching research methods in the social sciences (Lewthwaite and Nind, 2016). The combination of strategies, practices, and recommended software provides psychology academics with new avenues for teaching research methods and supporting student learning. As found by Allen and Baughman, and reported previously by Sizemore and Lewandowsky (2009), better outcomes do not necessarily equate with more positive attitudes toward research methods. These findings highlight the importance of addressing attitudes in addition to increasing students' knowledge and application of research methods.

## Author contributions

The author confirms being the sole contributor of this work and approved it for publication.

### Conflict of interest statement

The author declares that the research was conducted in the absence of any commercial or financial relationships that could be construed as a potential conflict of interest.
